# Tamil handwritten palm leaf manuscript dataset (THPLMD)

**DOI:** 10.1016/j.dib.2024.110100

**Published:** 2024-01-30

**Authors:** I. Jailingeswari, S. Gopinathan

**Affiliations:** Department of Computer Science, University of Madras, Chennai, India

**Keywords:** Tamil palm leaf, Enhancement, Binarization, Segmentation, Ground truth, Otsu, Photoshop

## Abstract

Most palm leaf manuscripts are generally accessible in deteriorated condition, including cracks, discoloration, moisture and humidity, and insects bite. Such a manuscript is considered challenging in the research field. We captured deteriorated Tamil palm leaves around 262 dataset samples are ‘Naladiyar(27)’,’ Tholkappiyam(221)’, and’ Thirikadugam(14)’ which are genned up mortal health, discipline, authoritative text on Tamil grammar. We contribute the high-quality raw dataset with the aid of a Nikon camera, pre-enhance samples by editing software tool, and applied the Otsu threshold to deliver the ground images through binarization as readily accessible content presenting a highly time-consuming task to play a vital role in Machine/Deep/ Transfer learning, AI, and ANN

Specifications Table

**Table:** 

Subject	Tamil handwritten palm leaf manuscript dataset (THPLMD)
Specific subject area	Tamil palm leaf manuscript enhancement, document binarization segmentation process.
Type of data	Table Image Figure
How the data were acquired	The dataset was captured in the sequence of five layers of palm leaf samples from each bundle placed on a white sheet that had been spread out on a table, facing the proper direction, under an appropriate lighting setup, to collect data. Using a DSLR camera like the NIKON D7200, manuscripts are collected and organized into three folders employing Photoshop editing software to tool for crop, resize and correct the dataset, and applied the Otsu threshold to make a ground truth image*.*
Data format	Degraded raw Tamil palm leaf manuscripts and binaries ground truth dataset in .jpg format.
Description of data collection	Collected palm leaf samples are in the condition under discoloration, cracks, staining, rodent activity, and insect bites. Handled the samples carefully and captured them at Dr. U.Ve.Swaminatha Iyer Library, Chennai, and Tamilnadu. The name of the samples are ‘NALADIYAR’ (27), ‘THOLKAPPIYAM’ (221), and ‘THIRIKADUGAM’ (14), Samples contain information on mortal discipline and health, authoritative text on Tamil grammar, and medicine.
Data source location	‘NALADIYAR’ (27), ‘THOLKAPPIYAM’ (221), ‘THIRIKADUGAM’ (14), from Dr.U.Ve.Swaminatha Iyer Library, Chennai. Tamilnadu, India.
Data accessibility	doi: 10.17632/xz9rx5wfc5.1

## 1 Value of the Data

A. Methodological Contribution:

• The dataset consists of a variety of linguistic units, including vowels, consonants, and compound characters, which facilitate the process of text mining. The disciplines of machine learning, deep learning, and transfer learning, as well as the specific areas of computer vision and image processing, are widely recognized in the academic context of artificial intelligence and artificial neural networks.
• The dataset collected comprises three distinct categories of writers, which were utilized as a testing dataset for the trained models, facilitating feature extraction and analysis in the domain of Tamil handwritten character recognition. The acquisition of readily available ground truth datasets poses a significant challenge to the advancement of learning models that entails significant time and resource investment for the research community.

B. Benefit of data:

The old ancient Tamil palm leaf manuscripts from Naladiyar, Tholkapiyam, and Thirikadugam as shown in Fig. 1a, Fig. 1b to Fig. 1(c) contain useful information that can benefit people.

• Naladiyar: (Four Hundred Quatrains):[7] It is composed of Jain monks, and deals with mortal morals and ethics, praising righteous conduct, highlighting the value of living a moral life, effective wealth management, and enjoyment.
• Tholkappiyam: (Ancient Tamil Grammar):[8] It is written by Tholkappiyar that discusses authoritative text on Tamil grammar, and literary topics as well as orthography, semantics, prosody, phonology, and morphology.
• Thirikadugam: (The Three Special Stimulants):[9] It is written by Nallathanar that adheres to secular ethics, the analogy to the traditional herbal medicine that treats stomach ailments with the three herbs sukku (dried ginger), milaku (pepper), and thippili (long pepper)

C. Reuse of data
  • The Tamil palm leaf dataset, which had old ancient days, was collected and photographed using a Nikon D7200 DSLR camera. This resulted in a high-quality standardized dataset that efficiently produces the ultimate binarized ground truth dataset that can be utilized for character-level modifications, exhibits are devoid of noise and degradation thereby providing a valuable resource for society in terms of facilitating visually perceptible and easily readable text.

**Fig. 1a:**
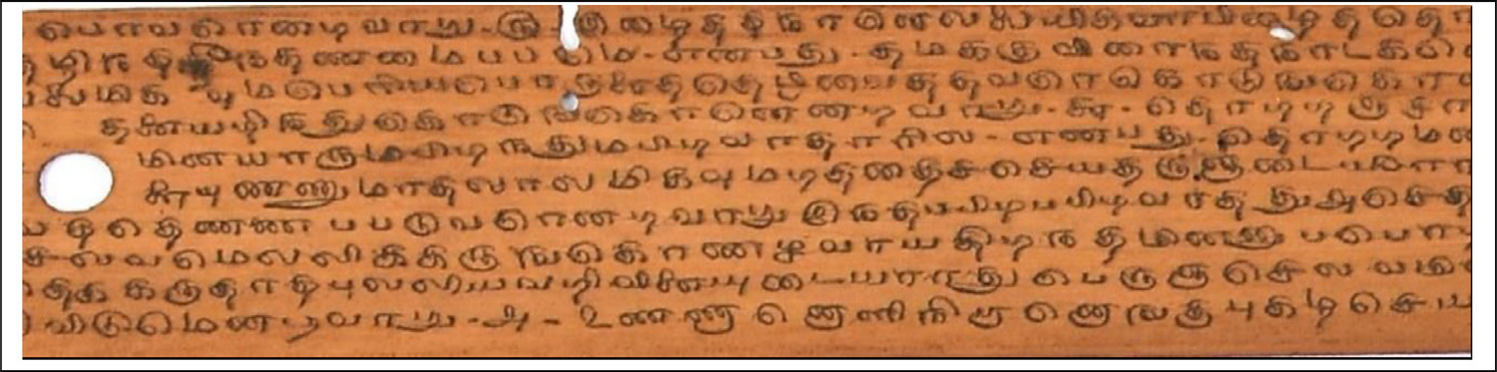
Tamil palm leaf samples of Naladiyar.

**Fig. 1b:**
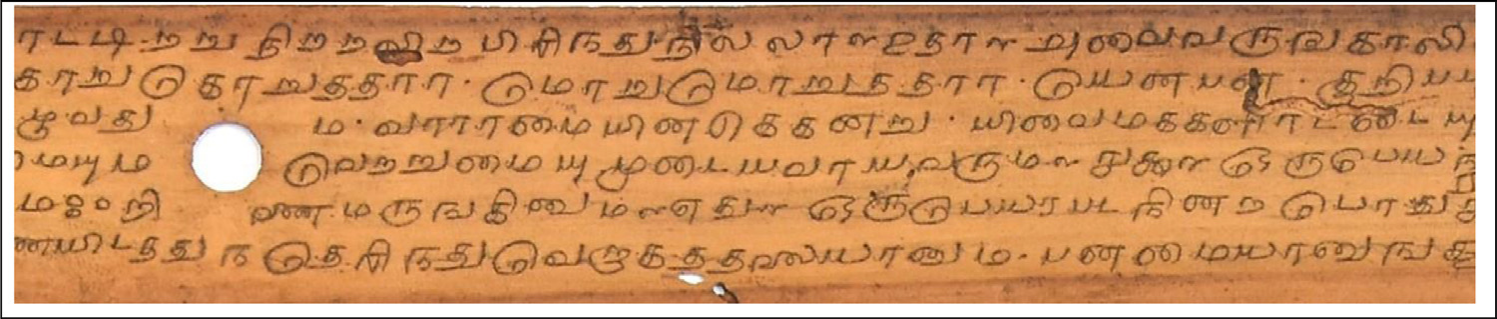
Tamil. palm leaf samples of Tholkappiyam

**Fig. 1c:**
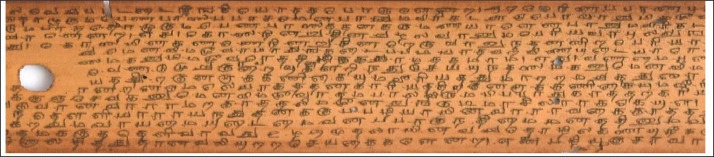
Tamil palm leaf samples of Thirikadugam

## 2 Objective

Palm-leaf manuscripts are considered one of the oldest and most widely spread methods utilized for this purpose by humanity with the transmission and preservation of writing in various cultures have been achieved by employing a variety of technologies. In India, particularly in the southern region, it is the most ancient form of written communication. The knowledge preserved in palm-leaf manuscripts proves to be highly valuable even for the current youth generation. Manuscripts tend to exhibit variation in size across distinct localities, with an average width of 4 centimeters and length of 48 centimeters, while also measuring more than 40 centimeters in thickness. Narayam was the primary tool to scribe on palm-leaf manuscripts called Thaliyola. In addition, palm leaf serves as the primary writing and drawing medium in countries like South and Southeast Asia, including Nepal, Sri Lanka, Burma, Thailand, Indonesia, and Cambodia. Manuscripts contain a wide range of information, including details about astrology, astronomy, and traditional medicines [1]. Documents and Manuscripts that contain cultural, historical and medical information about our rich and ancient culture cover a wide range of topics. The majority of these documents and Manuscripts are written on Palm leaves, which are susceptible to damage from handling, moisture, and fungus growth [2]. Due to the tropical climate of the area, the earlier palm leaf manuscripts have been completely destroyed. Climate, pollution, and biological factors like heat, moisture, humidity, discoloration, fungi, insect bites like silverfish and cockroaches, rodent activity, seepage of ink, smearing along the cracks, dirt, and other discoloration cause manuscripts to deteriorate [3].

**Fig. 2:**
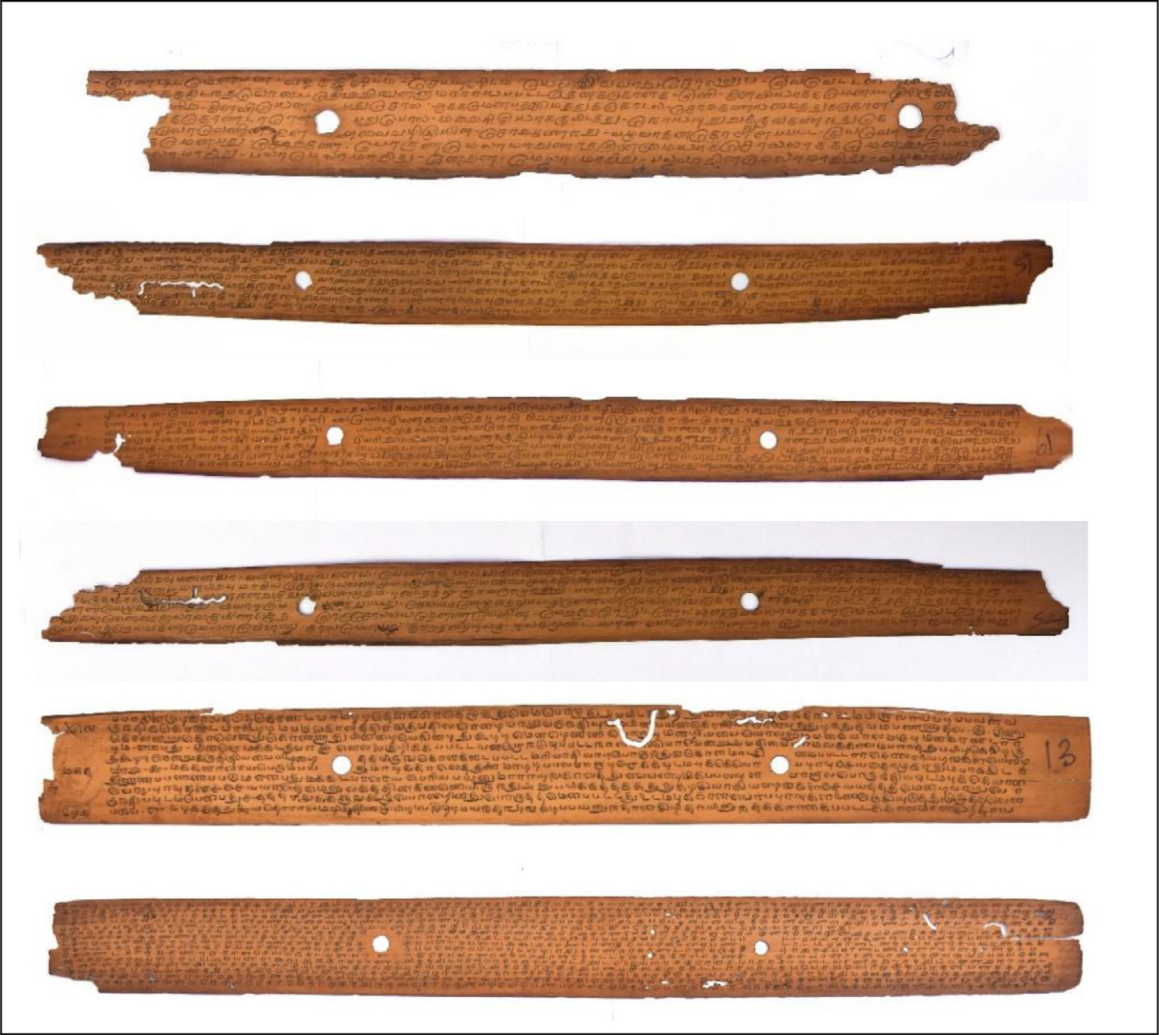
Degraded Tamil palm leaf samples.

The primary objective of the proposed work is to eliminate decay in the manuscript that avoids hard to understand text portions. Few degraded dataset samples obtained from the 'Naladiyar', 'Tholkappiyam', and 'Thirikadugam' are presented in Fig. 2. Which is composed of the authoritative text on Tamil grammar and medicine and is well-versed in promoting human health and discipline.

The development of a binarization method in the provided dataset is necessary for identifying and removing potential anomalies from being considered during data processing, therefore it reduces the probability of those abnormalities affecting any future post-processing procedures, thus ground truth dataset containing accurate information will aid in enhancing the performance of the Machine, Deep, Transfer Learning, as well as artificial intelligence and artificial neural network evaluation in the future.

## 3 Data Description

The captured raw data sets obtained from high-quality materials were used to ensure optimum efficiency in the final stage of ground truth binarization. Specifically, the degraded Tamil palm leaf manuscripts from Naladiyar, Tholkappiyam, and Thirikadugam were selected for their methodological advantages and potential for reuse, and are available in the repository. Photoshop software is used for editing dataset images to perform tasks such as cropping, resizing, and image correction. Then applied Otsu Threshold Algorithm to generate ground truth images. The Production of a binarized ground truth dataset is efficiently achieved through the utilization of high-quality standardized datasets with the help of a Nikon D7200 DSLR camera. The Proposed dataset comprised binarized ground truth without degradation or noise that will facilitate the general public to understand the text, such data sets are highly significant in the research community, specifically in the character-level modifications and progression of segmentation, enhancement, and feature extraction algorithms.

The dataset from the corresponding degraded Tamil palm leaf manuscript is described in Table 1 for accessibility. The dataset is made up of 27 accumulated samples from Naladiyar, 221 samples from Tholkappiyam, and 14 samples from Thirikadugam sourced from Dr. U. Ve Swaminatha Iyer Library in Tamilnadu. In total, there are 262 unprocessed images issn the dataset, 199 corresponding images with ground truth data are available at this link doi:10.17632/xz9rx5wfc5.1.

**Table 1:** Tamil palm leaf dataset collections.

Palm Leaf			
Description	Number of Original raw dataset images	Number of Ground Truth Dataset Images	Link Source
Naladiyar	27	26	doi:10.17632/xz9rx5wfc5.1
Tholkappiyam	221	163	doi:10.17632/xz9rx5wfc5.1
Thirikadugam	14	10	doi:10.17632/xz9rx5wfc5.1

## 4 Experimental Design, Materials, and Methods

### 4.1 Dataset Accumulation

Collected dataset samples of degraded Tamil palm leaf manuscripts are 262 in number under the condition of rodent exertion, humidity, and cracks. Accumulated samples from Dr.U.Ve.Swaminatha Iyer Library, Chennai, Tamilnadu, India, specifically the ‘Naladiayar’-27 samples, ‘Tholkappiyam’-221 samples, and ‘Thirikadugam’-14 samples as listed in Table 1. This manuscript serves multiple objectives, including providing details on human well-being, medicine, and authoritative literature on Tamil linguistic structures. The data repository containing all unprocessed raw sample datasets and binarized datasets can be readily accessed through an online platform. doi:10.17632/xz9rx5wfc5.1.

### 4.2 Dataset Acquisition

**Fig. 3:**
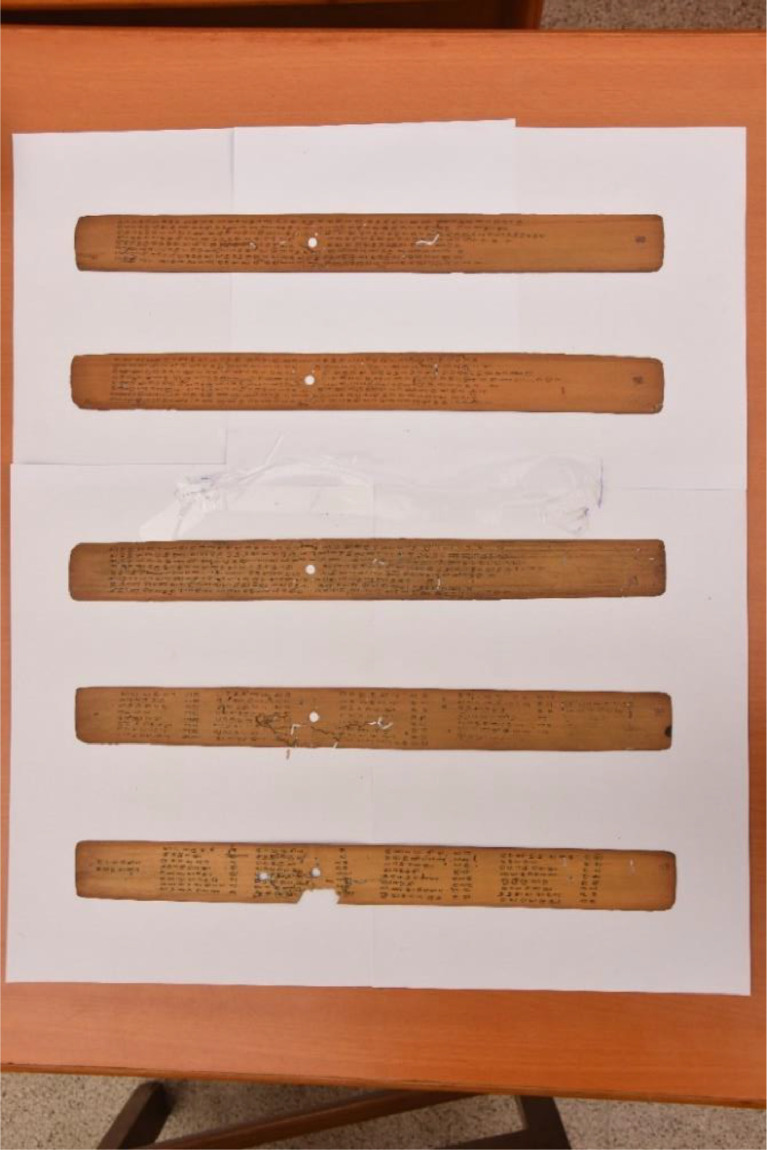
Technique for obtaining dataset.

The plain background plays an important role in the success of capturing the raw degraded palm leaf dataset, placed a white sheet on the table and on it 5 layers of palm leaves were handled carefully and arranged from bottom to top horizontally, then captured the palm leaf dataset using a Nikon D7200 DSLR camera to improve the quality of the image, with full focus attention of the viewer on it, set better angle position, facing the right way, and with the proper lighting setup to collect data are around 262 samples. An approach used to collect the dataset capturing method is shown in Fig. 3.

### 4.3 Dataset Preprocessing

As in getting raw palm leaf datasets that have been captured, using an acquisition tool to import such images, bring them into a digital system, and arranged datasets sequentially then visually analyzed whether clear or unblurred images appeared. Then the dataset segregates into three folders. The proposed method focuses on removing deterioration that obscures text section that employs Photoshop editing software performs basic editing tasks such as cropping, adjusting exposure, white balance and contrast removing blemishes, White balance (WB) the colour contrast are the process of removing unrealistic colour casts, so that image which appeared as neutral and is rendered white in the image.

Luminance and colour intensity to increase the brightness of the image, choosing a specific area to be considered a feather, then applying curve adjustments to the dataset image to balance the colours and correct the images. In order to create a ground truth image, the segmentation algorithm of the Otsu threshold [5] is then used. The implementation of the Otsu Threshold Algorithm resulted in the generation of ground truth images thus the selection of an appropriate threshold value is of utmost significance in this process [4]. The Otsu threshold value fixes the possible intensity value (t) for separating pixels into foreground (fg) and background (bg). Finding the minimum weighted variance between the fg and bg pixels until the process is iterated.

Lesser than the fixed value considers as foreground and greater than the fixed value is background. The formula weighted within the variance is given by Eq. (1) [6]

(1) $$\begin{array}{c} \sigma^{2}\left(t\right)=\Omega_{\mathrm{bg}}\left(t\right)\;{\sigma^{2}}_{\mathrm{bg}}\left(t\right)+\Omega_{\mathrm{fg}}\left(t\right)\;{\sigma^{2}}_{\mathrm{fg}}\left(t\right) \end{array}$$

**Fig. 4:**
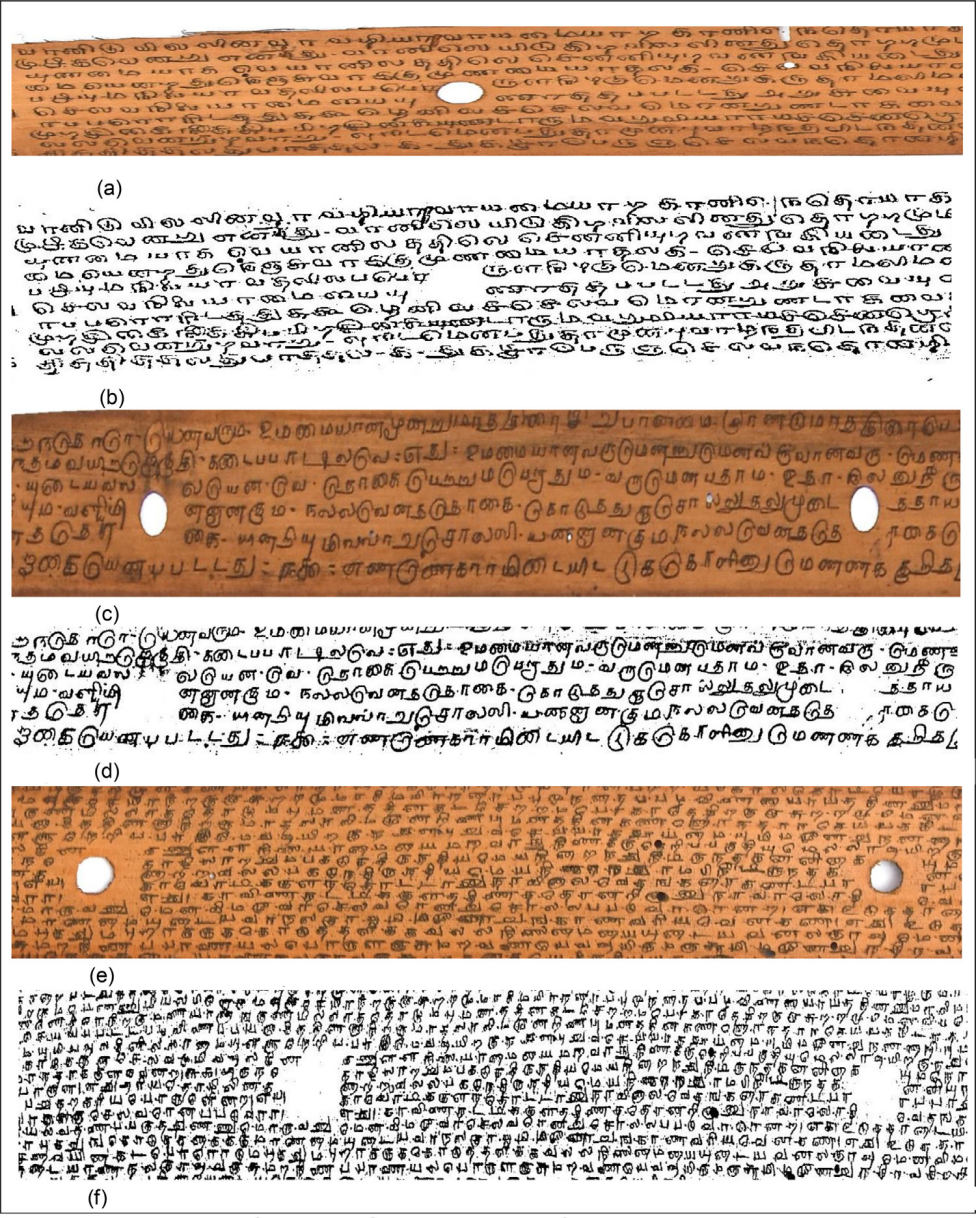
(a)–(f) Binarized Ground truth images for corresponding raw dataset images. (a) Original image of Naladiyar. (b) Binarized Ground truth image for the corresponding original image of Naladiyar Fig. 4(a). (c) Original image of Tholkappiyam. (d) Binarized Ground truth image for the corresponding original image of Tholkappiyam Fig. 4(c). e) Original image of Thirikadugam. f) Binarized Ground truth image for the corresponding original image of Thirikadugam Fig. 4 (e).

Where variance Ω_fg and_ Ω_bg_ are the probability of a number of pixels of foreground and background pixels at Threshold t, σ^2^ represents the variance of colour value. The key idea here is to iterate through all the possible values of the threshold and measure the spread of background and foreground pixels. Design the binarization technique to detect and filter possible imperfections from becoming the image for processing and potential cause of errors for post-processing steps. The ground truth dataset will facilitate future evaluation as saved in .jpg format. The Above Fig. 4(a)–(f) shows the experimental result in the order of the original image and binarized ground truth images for the corresponding original image.

## Ethics Statements

This work does not involve human subjects and animals experiments.

## CRediT authorship contribution statement

**I. Jailingeswari:** Conceptualization, Formal analysis, Investigation, Data curation, Methodology, Resources, Writing – review & editing, Writing – original draft, Validation. **S. Gopinathan:** Conceptualization, Formal analysis, Investigation, Data curation, Methodology, Resources, Writing – review & editing, Writing – original draft, Validation, Supervision.

## Data Availability

Tamil Palmleaf Dataset (Original data) (Mendeley Data).
